# Impact of midwife-led antenatal care on childbirth outcomes and maternal satisfaction among low-risk mothers in India

**DOI:** 10.6026/973206300215000

**Published:** 2025-12-15

**Authors:** Revathi Ramasamy, Lilly Christopher, Shankar Shanmugam Rajendran, Jeyabharathi B, Nirmala A, Winston Thomas S

**Affiliations:** 1 Department of Obstetrics & Gynaecological Nursing, Meenakshi Academy of Higher Education and Research, MAHER (DU) & NPME, College of Nursing, Madras Medical College, Chennai, India; 2 Department of Medical and Surgical Nursing, Meenakshi Academy of Higher Education and Research, MAHER (DU), Chennai, Tamil Nadu, India; 3 Department of Child Health Nursing, College of Nursing, Madras Medical College, Affiliated to The Tamil Nadu Dr MGR Medical University, Chennai, India; 4 Department of Obstetrics & Gynecology, Institute of Obstetrics & Gynecology, Chennai, India; 5 Department of Paediatrics, Institute of Child Health & Hospital for Children, Egmore, Chennai, India

**Keywords:** Antenatal care, birth outcomes, midwife-led care, neonatal outcomes, satisfaction

## Abstract

Our understanding of midwife-led antenatal care by demonstrates its positive impact on maternal satisfaction and favorable birth outcomes in low-risk women essential. Therefore, it is of interest to evaluate the impact of midwife-led antenatal care for low-risk women at a tertiary hospital. The objectives were to assess maternal and neonatal outcomes and explore how midwife-focused education influences maternal satisfaction. The study involved 150 low-risk primiparous women, using structured questionnaires and clinical records. Results showed high rates of spontaneous vaginal birth (71.33%) and positive maternal satisfaction, with 78% of mothers reporting no distress during labor. Thus, we show the need for evaluating the effectiveness of midwife-led antenatal care in improving maternal and neonatal outcomes for low-risk women at a tertiary hospital.

## Background:

Midwifery-lead care has been developed as an evidence- based model of maternity care that focuses on supporting the philosophy for natural birth and women-centred care [1]. This method is based on the philosophy that pregnancy and childbirth should occur with little or no interference, allowing women to complete the birthing process naturally [2]. The model of midwifery is predicated on research that has demonstrated the efficacy of care to improve pregnancy and birth outcomes as well as mothers' satisfaction. This model highlights the value of educating women, promoting a sense of control and avoiding over- medicalization [3]. Midwife antenatal care is the care that a midwife who has undergone some formal training provides and monitor health status of low-risk pregnant women during pregnancy, labour and postpartum period. Low-risk mothers are those without any major obstetric or medical complications throughout their pregnancy and whose births took place between 37 to 41 weeks gestation [4]. This type of care has been found to facilitate a more individualised approach to the birthing process, allowing women control, guidance and support from their midwife and a developing relationship of trust [5]. In the present study, the impact of midwife-led care will be assessed by looking at a range of maternal outcomes including: spontaneous vaginal birth; intranatal complications and mothers' overall satisfaction with their birth experience. Neonatal events, further such as birth weight and rate of pre-term births or birth asphyxia will also be evaluated [6,7]. Its purpose is to address whether care involves a midwife living principles of physiologic childbirth and birth will optimize the outcomes for low-risk women [8]. Therefore, it is of interest to investigate the effect of midwife-led education on maternal satisfaction and the woman's experience of childbirth.

## Methodology:

This article has used a quantitative research method to achieve an objective understanding of the influence of midwife-led antenatal care on child birth outcomes and maternal satisfaction. The quantitative method enabled data to be collected and analyzed in a numerical form with the aim being to determine trends or patterns on which make generalizations. It was an observational study in which the results and maternal experiences of low-risk women receiving care by independent midwives were observed and written down without any intervention as to what factors were considered. This methodology has been right for reading the unintended consequences and relationships of factors in the current health service. The research was carried out in a tertiary care hospital, offering midwife-led antenatal care. There were midwifery clinic, delivery room with midwives trained and so the area was perfect to do the study. The study population was low-risk pregnant women visiting the midwife-led antenatal care at the tertiary care hospital. Mothers at low-risk of complications were those who had no comorbid medical, obstetric or psychiatric conditions during pregnancy and were anticipated to have a normal labor and delivery. A total of 150 low-risk mothers were included in the present study. Sample size was decided to ensure adequate number of data for analysis and feasibility within the scope of study. The purposive sampling technique was used in choosing the participants for the research. This sampling was justified as it concentrated on a particular cohort of mothers that met the inclusion criteria of attending midwife-led antenatal care. This enabled the sample to be representative of the target population low risk mothers receiving care from midwives. The following inclusion criteria were used in our experiment: Low risk pregnant women who had made a minimum of three antenatal visits after 28 weeks gestation. Females, who could read, speak and understand Tamil or English. Participants Women who showed interest in the research and signed an informed consent form.

## Exclusion criteria:

Women were ineligible if one of the following was present: They belonged to other studies. They had complications during pregnancy or labor that increased their risk (e.g., pregnancies for which multidisciplinary care was needed). They could neither understand nor converse in Tamil or English. The information was obtained through structured questionnaires and clinical files. The following methods were used the most for gathering information: Maternal satisfaction questionnaire: This was used to assess the extent of satisfaction with antenatal, labour and postnatal care provided by midwives. Variables related to emotional support, staff contact, sense of control during delivery and general experience were included in the questionnaire. Clinical outcomes sheet: This provided information such as the type of delivery (vaginal delivery or caesarean section), presence of complications, birth weight and neonatal results.

## The main variables of the study were:

## Maternal outcomes:

[1] Manner of delivery (spontaneous vaginal birth, cesarean section, instrumental vaginal birth).

[2] Occurrence of any problem during delivery (example; intranatal problems).

[3] Episiotomy and perineal tear occurrence.

[4] Maternal satisfaction levels.

[5] Neonatal Outcomes:

[6] Birth weight (low birth weight [LBW], normal birth weight, high birth weight).

[7] Incidence of preterm birth.

[8] Neonatal morbidities (asphyxia, NICU admissions).

Data was analyzed through descriptive and inferential statistics. Statistics descriptive (i.e., sample means, percentages and standard deviations) were employed to summarize the sample characteristics and study outcomes. Associating maternal demographic variables (age, number of visits), and birth outcomes or level of maternal satisfaction was tested using inferential statistical tests such as Chi-square or t-test. The experiment was ethically reviewed and approved. The participants were explained with the objective of the study and written consent was taken for further data collection. Anonymity was preserved through anonymization of the subject data, and collected data were kept safely. Participants had the right to stop participation at any stage of the study with no consequences. Participating low-risk mothers were recruited at a midwife-led care activity in a tertiary level hospital and study results may not be transferable to high-risk populations or individuals receiving care in various other healthcare settings. Moreover, the fact that the satisfaction was self-reported could have inevitably led to subjective biases in the data. The effectiveness of midwife-led antenatal clinic services in influencing childbirth outcomes and maternal satisfaction among mothers at a tertiary care hospital has not been fully explored. While midwifery care is often associated with improved birth outcomes, particularly for low-risk mothers, the specific impacts on maternal satisfaction and the overall birth experience remain to be comprehensively assessed. This study seeks to evaluate the role of midwife-led care in improving maternal and neonatal outcomes, as well as enhancing the overall satisfaction of mothers with their birth experience. Revised birth satisfaction scale is shown in Table 1 (see PDF).

## Results:

The study assessed key outcomes of midwife-led antenatal care among low-risk mothers. The sample comprised 150 low-risk mothers. The findings are summarized in the following sections: In terms of the age distribution, 64.67% (97 mothers) were aged 21 to 25 years. While 22.67% (n = 34) of the mothers were 26-30 years old, only 7.33% (n = 11) were aged below the age of twenty and5.33% (n =8) were above thirty years old. In the obstetric history, 101 (67.33%) mothers were non-booked and had no history of normal delivery; at the same time, 49 mothers (32.67%) were booked with a previous history of normal delivery. With respect to number of antenatal visits, the majority (77.33%, 116 mothers) received less than three visits while 22.67% (34 mothers) had more than three visits. In terms of mode of birth, the majority (71.33%, 107 mothers) had spontaneous vaginal birth (SVB), while 26.00% (39 mothers) underwent LSCS and 2.67% (4 mothers) had AVB. 136 mothers (88.67%) delivered at KGH and the place of delivery was mainly at the tertiary hospital KGH. Smaller proportions also delivered elsewhere: 4.67% (7 mothers) at MCH/DCH, 1.33% (2 mothers) at primary-level hospital and 5.33% (8 mothers) in private facilities. Regarding episiotomy, 69.33% (104 mothers) were cut and 30.67% (46 mothers) did not undergo this procedure. Only 3.33% (5 mothers) reported perineal tear and 96.67% (145 mothers) did not. As for birth weight, greater number of babies also fell between 2.5 to 3.0 kg (53.33%, 80 babies) and less than 34.67% (52 babies) weighed less than 2.5 kg. On 12.00% (18 babies) were heavier than 3.0 kg. Intranatal complications were recorded in 14.67% (22 mothers) and not experienced by 85.33% (128 mothers). Gestational age (GA) at birth indicated that 92.67% of babies (n = 139) were born full-term (>37 weeks), with smaller proportions delivered earlier, namely, 2.67% (<28 weeks; n = 4), 2.67% (28-32 weeks; n = 4), and 2.00% (33-37 weeks; n = 3). NICU admission rates were 32.67% (49 babies) and not admitted 67.33% (101 babies). The proportion starting BF was 68.67% (103 mothers) and the one that did not start BF was 31.33% (47 mothers). These results are summarized in Table 2 (see PDF).

The study also assessed birth satisfaction using six statements. The results showed high satisfaction levels across several areas of the birth experience: Regarding the statement, "I was not distressed at all during labor," 78.00% (117 mothers) agreed, while 19.33% (29 mothers) somewhat agreed and only 2.67% (4 mothers) disagreed. On the statement, "I felt very anxious during my labor and birth," the majority of mothers (52.00%, 78 mothers) disagreed, and 47.33% (71 mothers) somewhat agreed. Only 0.67% (1 mother) agreed. When asked, "I felt well supported by staff during my labor and birth," 48.67% (73 mothers) strongly agreed, and another 48.67% (73 mothers) somewhat agreed. Only 2.67% (4 mothers) disagreed. For the statement, "I found giving birth a distressing experience," 50.67% (76 mothers) somewhat disagreed, while 41.33% (62 mothers) disagreed, and 8.00% (12 mothers) agreed. Regarding the statement, "I felt out of control during my birth experience," 51.33% (77 mothers) somewhat disagreed, and 44.67% (67 mothers) disagreed, with only 4.00% (6 mothers) agreeing. Finally, regarding "The staff communicated well with me during labor," 56.00% (84 mothers) strongly agreed, and 44.00% (66 mothers) somewhat agreed, with no mothers disagreeing. These results, illustrating the overall high satisfaction with midwife-led care, are presented in Table 3 (see PDF). Figure 1 (see PDF) shows the distribution of gestational age (GA) at birth among the women surveyed. A significant majority, 92.67%, gave birth after 37 weeks of pregnancy. A smaller percentage of women delivered between 33-37 weeks (2%), and even fewer delivered between 28-32 weeks (2.67%). Very few women delivered before 28 weeks, accounting for just 2.67% of the cases.

## Level of birth satisfaction score:

The Figure 2 (see PDF) highlights the birth satisfaction scores of the mothers. The majority (64.67%) of mothers reported a high level of satisfaction with their birth experience. A smaller group, 35.33%, indicated a moderate level of satisfaction, while none of the mothers reported a low satisfaction score. Figure 3 (see PDF) explores how the mode of delivery influences satisfaction. For mothers who had a spontaneous vaginal birth (SVB), 70.09% reported high satisfaction, while 29.91% were moderately satisfied. In contrast, 51.28% of mothers who had a cesarean section (LSCS) reported high satisfaction, while 48.72% reported moderate satisfaction. Finally, for assisted vaginal birth (AVB), 50% of mothers reported both high and moderate levels of satisfaction.

## Discussion:

This study's results are consistent with the literature supporting the beneficial effect of midwife-led antenatal care on maternal and neonatal outcomes. The findings reveal high rates of spontaneous vaginal delivery, good birth weights and low complication rates as previously reported. The study showed spontaneous vaginal births (SVB) 71.33%, LSCS 26.00% and AVB 2.67%. These rates are similar to those found in other studies. For example, the work of Sriram *et al.* (2024) [1] studied the role of NFL in both axonal damage and regeneration, using a two sides-over hemisphere experimental design and identified that midwife-led care decreased interventions in childbirth and was associated with positive maternal outcomes. Likewise, a systematic review by Fikre (2023) [9] also reported better maternal outcomes of women in low-risk pregnancies being higher via midwifery-led care than other types of perinatal health care delivery. As for neonatal outcomes, our study showed the low ncidence of preterm delivery (7.33%) and high percentage of full-term birth (92.67%). The distribution of birth weight: 53.33% of all babies weighed between 2.5-3 kg and >3 kg was for only 12.00%. These results are in partial agreement with those reported by Ramu *et al.* (2025) [10], which indicated that midwife-led care might lead to better maternal and fetal outcomes than standard obstetrician led-care would. Maternal satisfaction was also measured, and nearly 80.00% "strongly agreed" for the statement "I did not feel upset at all during labour", while half of respondents (56.00%) "Somewhat agreed" for "staffs communicated with me well during my labour". These results correspond to a Wanyenze *et al.* (2023) [11], who observed higher satisfaction with labor among women in the intervention period compared to the control group. In comparison to other studies, the results of this study are in line with international findings. For example, a research in Iran by Sighaldeh *et al.* (2025) [12] demonstrated that midwifery-led care achieved better maternal and neonatal outcomes compared with usual care. Furthermore, research conducted by Hailemeskel *et al.* Lead maternity care is provided by a midwife who remains with the birth party, providing all necessary services, as appropriate or applicable. Hailemeskel (2022) [13] reported that midwifery continuity of care increased women's satisfaction with maternity care among low-risk women. All efforts matter although the study is insightful, there are limitations that need to be recognized. The sample may neither be representative of the population as a whole nor standard for other centers due to this small size (only 150 low-risk mothers who delivered at a single tertiary care hospital). Besides, confounders like socioeconomic status, education and culture which may impact on the results were not taken into consideration by the study.

## Conclusion:

Finally, this study contributes to the increasing body of evidence that midwife-led antenatal care is beneficial for maternal and child outcomes. The high rate of spontaneous vertex delivery, excellent parity distribution and birth weight profile, the low complication rate, as well as good maternal satisfaction with midwifery care serve to affirm these values. Larger sample size and more mixing populations are suggested for the future research together with more factors to be taken into evaluation order to enhance the evidence base for health policy.
